# The role of MAFLD in predicting major adverse cardiovascular events in patients with stable COPD: a prospective cohort study

**DOI:** 10.3389/fmed.2026.1793125

**Published:** 2026-05-25

**Authors:** Le Minh Hanh Doan, Thuong Vu Le, Thi Khanh Tuong Tran

**Affiliations:** 1 Internal Medicine Department, Pham Ngoc Thach University of Medicine, Ho Chi Minh City, Vietnam; 2 Internal Medicine, University of Medicine and Pharmacy at Ho Chi Minh City, Ho Chi Minh City, Vietnam

**Keywords:** chronic obstructive pulmonary disease, COPD, MACE, MAFLD, major adverse cardiovascular events

## Abstract

**Background:**

Cardiovascular disease is a major cause of morbidity and mortality in patients with chronic obstructive pulmonary disease (COPD). Metabolic dysfunction–associated fatty liver disease (MAFLD) is highly prevalent in COPD and reflects shared cardiometabolic and inflammatory pathways. However, its role in predicting cardiovascular outcomes within COPD populations remains insufficiently characterized.

**Methods:**

We conducted a prospective cohort study involving 168 patients with stable chronic obstructive pulmonary disease, with continued follow-up from a previously published report in the Vietnamese Journal of Medicine. Baseline MAFLD was defined using transient elastography in combination with metabolic criteria. Patients were followed for 12 months for the occurrence of first major adverse cardiovascular events (MACE). The analysis was structured as (1) an exposure-based analysis evaluating the association between MAFLD and MACE, and (2) an exploratory prognostic modeling approach using Bayesian Model Averaging, with model performance evaluated in terms of discrimination, calibration, clinical utility, and nomogram-based risk estimation.

**Results:**

During follow-up, 24 patients (14.3%) experienced MACE. MAFLD was significantly more frequent in patients with MACE than in those without (79.2% vs. 43.8%). In survival analysis, MAFLD was associated with shorter MACE-free survival and a more than fourfold increased risk of incident events. In Bayesian analyses, MAFLD showed the highest posterior inclusion probability, followed by dyspnoea severity assessed by the modified Medical Research Council scale. A multivariable model incorporating MAFLD, mMRC, and high-sensitivity C-reactive protein showed fair-to-acceptable discrimination (AUC ≈ 0.75) with internal calibration assessment and was used to derive an exploratory nomogram for 12-month MACE risk estimation.

**Conclusion:**

In patients with stable COPD, MAFLD is a strong and consistent predictor of 12-month MACE. These findings suggest that cardiovascular risk stratification in COPD populations may benefit from incorporating cardiometabolic comorbidities, particularly MAFLD. A Bayesian model incorporating MAFLD, mMRC, and hs-CRP showed preliminary discriminatory ability for 12-month cardiovascular risk stratification, but requires external validation before clinical use.

## 1 Introduction

Chronic obstructive pulmonary disease (COPD) is a major global public health condition characterized by persistent respiratory symptoms and progressive airflow limitation. According to the Global Initiative for Chronic Obstructive Lung Disease (GOLD) 2025, COPD remains one of the leading causes of morbidity and mortality worldwide, with a continuously increasing burden driven by population aging, sustained exposure to tobacco smoke and air pollution, and the rising prevalence of cardiometabolic comorbidities (1). Although COPD is preventable and treatable, it is frequently underdiagnosed and undertreated, particularly in low- and middle-income countries (LMICs), where most patients present at more advanced stages of disease (1, 2).

Beyond respiratory impairment, COPD is increasingly recognized as a systemic disease with a high burden of comorbid conditions. Among these, cardiovascular disease (CVD) is the most prevalent and clinically significant. Patients with COPD have a markedly increased risk of major adverse cardiovascular events (MACE), including myocardial infarction, stroke, heart failure, and death (3, 4). The coexistence of COPD and other disease substantially amplifies healthcare utilization, hospitalization rates, and economic burden. In LMICs both direct and indirect costs impose a major societal burden due to reduced productivity, disability, and limited access to effective long-term care. GOLD 2025 emphasizes that outcomes in COPD are strongly influenced by comorbidities rather than airflow limitation alone, underscoring the need for integrated risk assessment strategies (1).

Despite this, cardiovascular risk in COPD is frequently underestimated in routine clinical practice (5). Traditional cardiovascular risk scores were developed in general populations and may not adequately capture COPD-specific pathophysiology or inflammation-related biomarkers (6). Therefore, there is a critical need for a specific prediction models to identify patients with stable COPD at high risk of MACE. Bayesian Model Averaging (BMA) provides a principled framework to address model uncertainty by integrating information across multiple plausible models and weighting predictors according to their posterior inclusion probabilities. Applying BMA to MACE prediction in stable COPD may improve predictive performance, enhance model robustness, and support personalized preventive strategies, aligning with the precision medicine approach advocated by GOLD 2025 (1, 7).

## 2 Materials and methods

### 2.1 Study design

This study was designed as a prospective cohort study in a previously characterized population of 168 patients with stable chronic obstructive pulmonary disease (COPD). This population was derived from an earlier cross-sectional study on metabolic dysfunction–associated fatty liver disease (MAFLD) conducted at the Asthma–COPD Management Unit of Nhan Dan Gia Dinh Hospital, Ho Chi Minh City, Vietnam, between June 2023 and June 2024, the baseline results of which were published previously in Vietnam Journal of Medicine (2025) (doi: https://doi.org/10.51298/vmj.v546i2.12661) (8). In the present study, all eligible participants from that baseline population were followed for 12 months, during which incident major adverse cardiovascular events (MACE) were prospectively ascertained.

### 2.2 Study aim

The primary aim of this study was to examine the association between baseline MAFLD and incident 12-month major adverse cardiovascular events in patients with stable COPD.

The secondary aim was to develop an internally evaluated prognostic model for 12-month MACE risk using baseline clinical variables. To reflect these aims, the analysis was structured in two complementary components: (1) an exposure-based analysis, in which MAFLD status was evaluated in relation to incident MACE and time-to-event outcomes; and (2) a prognostic modeling analysis, in which MAFLD was included as a prespecified candidate predictor alongside other clinical variables.

### 2.3 Participants

The study population consisted of *stable COPD patients* who had been included in the original investigation and fulfilled all predefined eligibility criteria at baseline.

Inclusion Criteria: Patients were included in the original study if they were diagnosed with stable COPD according to the Global Initiative for Chronic Obstructive Lung Disease (GOLD 2023) recommendations (9), attended regular follow-up at the outpatient Asthma–COPD Management Unit of Nhan Dan Gia Dinh Hospital during the study period, and provided written informed consent to participate.

Exclusion Criteria: The exclusion criteria applied in the original study were retained for the current analysis. Patients were excluded if they met any of the following conditions:

- Inability to undergo liver stiffness measurement by transient elastography (FibroScan®) due to central obesity or a body mass index (BMI) > 30 kg/m^2^.
- Unreliable FibroScan® measurements, defined as an interquartile range to median ratio (IQR/median) > 30% or a success rate < 60%.
- Evidence of intrahepatic or extrahepatic cholestasis, as identified by abdominal ultrasonography performed prior to FibroScan® assessment.
- Acute hepatitis, defined as serum aspartate aminotransferase (AST) and/or alanine aminotransferase (ALT) levels exceeding five times the upper limit of normal (35 U/L for men and 25 U/L for women).
- Pregnancy or breastfeeding at the time of enrollment.

No additional exclusion criteria were applied during follow-up.

All 168 participants met these criteria at baseline and were included in the longitudinal analysis.

### 2.4 Procedures

Baseline clinical assessment, laboratory testing, spirometry, and hepatic evaluation were collected at study entry as described in the original publication. Hepatic steatosis was assessed using transient elastography with controlled attenuation parameter (CAP). Participants were subsequently followed for 12 months through scheduled outpatient visits, review of hospital medical records, and structured telephone interviews.

The primary exposure was metabolic dysfunction–associated fatty liver disease (MAFLD), defined according to the Asia–Pacific consensus criteria (2020, updated 2024–2025) (10, 11). MAFLD was diagnosed by the presence of hepatic steatosis in FibroScan (CAP >234 dB/m) in combination with overweight/obesity (BMI ≥ 23 kg/m^2^ for Asians), or type 2 diabetes mellitus, or at least two metabolic risk abnormalities in individuals with normal body weight, including increased waist circumference, elevated blood pressure or antihypertensive treatment, hypertriglyceridemia, reduced HDL-cholesterol, prediabetes, insulin resistance (HOMA-IR > 2.5), or elevated high-sensitivity C-reactive protein (>2 mg/L) (12). MAFLD was analysed as a binary variable (present vs. absent).

### 2.5 Outcomes

The primary outcome was the first occurrence of major adverse cardiovascular events (MACE) during follow-up, defined as a composite of (13):

- Acute myocardial infarction, diagnosed according to the Fourth Universal Definition of Myocardial Infarction (14).
- Coronary revascularization (percutaneous coronary intervention or coronary artery bypass grafting);
- Hospitalization for heart failure was defined as an unplanned hospital admission due to new-onset or worsening heart failure requiring urgent evaluation and treatment. Heart failure–related hospitalization was classified into four clinical phenotypes: (1) acute *de novo* heart failure, defined as first presentation without prior heart failure; (2) acute decompensated chronic heart failure, representing worsening of known heart failure; (3) acute pulmonary oedema, characterized by sudden severe dyspnoea due to pulmonary congestion; and (4) cardiogenic shock, defined by primary cardiac pump failure with hypotension and tissue hypoperfusion (15).
- Stroke was defined as an acute neurological deficit of vascular origin, confirmed by neuroimaging, and classified as ischemic or hemorrhagic stroke (16).
- All-cause mortality.

Events were identified from hospital records or discharge documentation and adjudicated by investigators blinded to baseline MAFLD status.

### 2.6 Statistical analysis

Candidate predictors were prespecified based on clinical relevance and prior evidence, including age, comorbidity burden, the modified Medical Research Council (mMRC) dyspnoea scale, metabolic dysfunction–associated fatty liver disease (MAFLD), high-sensitivity C-reactive protein (hs-CRP), and high-sensitivity troponin I (troponin I-hs). All 168 patients with stable COPD formed the baseline cohort, in whom MAFLD status was determined at study entry, and were followed for 12 months for prospective ascertainment of incident major adverse cardiovascular events (MACE). Outcome-based comparisons were conducted according to MACE status, time-to-event analyses according to baseline MAFLD status, and exploratory prognostic modeling using Bayesian Model Averaging (BMA). BMA was implemented within a logistic regression framework to account for model uncertainty. Posterior model probabilities were estimated using the Bayesian Information Criterion, and posterior inclusion probabilities were calculated for each predictor. Effect estimates were expressed as posterior mean odds ratios with 95% credible intervals derived from the Bayesian model ensemble.

The highest posterior probability model (HPM) was first identified as the model with the largest posterior probability. However, given that the HPM may be overly parsimonious and potentially limit clinical applicability, an additional selection step was performed. Among Bayesian-supported models, discriminative performance was assessed using the area under the receiver operating characteristic curve (AUC). The final model for nomogram development was selected based on the highest AUC while remaining within the Bayesian model space. This strategy balances Bayesian evidence with predictive performance and clinical interpretability. Model performance was evaluated through discrimination which was assessed using ROC analysis and quantified by the AUC.

Calibration was evaluated using: bootstrap calibration curves (200 resamples) generated with the rms package; calibration intercept and slope, obtained from logistic recalibration of observed outcomes on predicted log-odds; mean absolute error (MAE) and mean squared error (MSE) between predicted and observed risks.

Clinical utility was assessed using Decision Curve Analysis (DCA), estimating net benefit across a range of threshold probabilities and comparing the selected model with “treat-all” and “treat-none” strategies.

A nomogram was constructed to estimate individual risk of MACE, mapping predictor values to total points and corresponding predicted probabilities.

Ethical approval for the original study and the follow-up analysis was obtained from the institutional review board of Nhan Dan Gia Dinh Hospital. All participants provided written informed consent prior to enrollment.

## 3 Results

**Figure 1:**
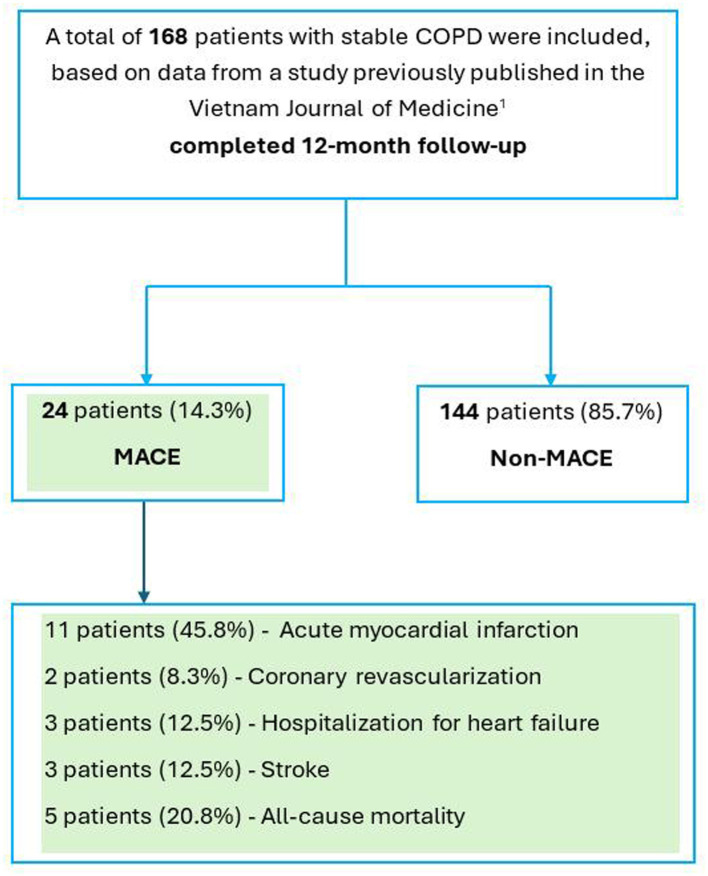
Study flow chart.

A total of 168 patients with stable COPD completed 12-month follow-up. Major adverse cardiovascular events (MACE) occurred in *24 patients (14.3%)*, including acute myocardial infarction, coronary revascularization, hospitalization for heart failure, stroke, and all-cause mortality (Figure 1).

Among patients who developed MACE, acute myocardial infarction was the most frequent event, accounting for 45.8% of all MACE cases. Coronary revascularization occurred in 2 patients (8.3%). Hospitalization for heart failure was observed in 3 patients (12.5%). Stroke occurred in 3 patients (12.5%). All-cause mortality was recorded in 5 patients (20.8%).

Patients who developed MACE had significantly higher symptom burden, as reflected by a higher mean COPD Assessment Test (CAT) score compared with those without MACE (19.9 ± 5.5 vs. 17.1 ± 5.4, *p* = 0.018). In addition, a history of frequent exacerbations (≥2 moderate or severe exacerbations in the preceding year) was more common in the MACE group (58.3% vs. 34.7%, *p* = 0.040). The distribution of GOLD symptom–risk groups differed significantly between groups (*p* = 0.044), with a higher proportion of patients classified as GOLD group E among those who experienced MACE (75.0% vs. 47.2%). In contrast, no significant differences were observed between the MACE and non-MACE groups with respect to age, sex, smoking status, body mass index, waist circumference, disease duration, baseline dyspnoea severity (mMRC ≥ 2), lung function parameters (FEV₁ in litres and percent predicted), airflow limitation severity, use of inhaled corticosteroids, medication adherence, or the presence of comorbidities (all *p* > 0.05) (Table 1).

**Table 1:** Baseline clinical characteristics according to the occurrence of major adverse cardiovascular events (MACE).

Characteristics	Total (*n* = 168)	MACE (*n* = 24)	Non-MACE (*n* = 144)	*p* value
Age, years	68.3 ± 8.9	69.7 ± 10.9	68.1 ± 8.6	0.411^€^
Male sex, *n* (%)	150 (89.3)	23 (95.8)	127 (88.2)	0.475^‡^
Body mass index, kg/m^2^	21.2 ± 3.5	22.2 ± 3.2	21.0 ± 3.5	0.129^€^
Waist circumference, cm	86.1 ± 11.8	90.1 ± 10.9	85.5 ± 11.9	0.077^€^
Current or former smoker, *n* (%)	149 (88.7)	24 (100)	125 (86.8)	0.210^‡^
Disease duration, years	5.0 (3.0–8.0)	5.5(5.0–10.0)	5.0 (3.0–8.0)	0.071^§^
mMRC ≥ 2, *n* (%)	141 (83.9)	23 (95.8)	118 (81.9)	0.131^‡^
CAT score	17.5 ± 5.5	19.9 ± 5.5	17.1 ± 5.4	**0.018** ^€^
Frequent exacerbations^a^, *n* (%)	64 (38.1)	14 (58.3)	50 (34.7)	**0.040** ^†^
GOLD group, *n* (%)				
A	23 (13.7)	1 (4.2)	22 (15.3)	**0.044** ^**‡**^
B	59 (35.1)	5 (20.8)	54 (37.5)	
E	86 (51.2)	18 (75.0)	68 (47.2)	
Maintenance therapy including ICS, *n* (%)	118 (70.2)	17 (70.8)	101 (70.1)	1.000^†^
Morisky adherence score	6.8 ± 0.9	6.7 ± 1.1	6.8 ± 0.9	0.664^€^
Presence of comorbidities, *n* (%)	125 (74.4)	20 (83.3)	105 (72.9)	0.325^‡^
FEV1 (L)	1.4 ± 0.5	1.2 ± 0.5	1.4 ± 0.5	0.102^€^
FEV1 (% predicted)	62.2 ± 20.7	56.3 ± 20.5	63.2 ± 20.6	0.127^€^
Airflow limitation severity, *n* (%)				
GOLD 1	34 (20.2)	3 (12.5)	31 (21.5)	0.071^‡^
GOLD 2	85 (50.6)	9 (37.5)	76 (52.8)	
GOLD 3	47 (28.0)	11 (45.8)	36 (25.0)	
GOLD 4	2 (1.2)	1 (4.2)	1 (0.7)	

Data are presented as mean ± SD, median (interquartile range), or *n* (%), as appropriate.

^†^*p* value calculated using the Chi-square test.

^‡^*p* value calculated using Fisher’s exact test.

^§^*p* values calculated using Mann–Whitney *U* test.

^€^*p* values calculated using Student’s *t*-test.

a Frequent exacerbations defined as ≥2 moderate or severe exacerbations in the previous year.

Bold values indicate statistically significant *p* values (*p* < 0.05).

Laboratory and hepatic characteristics stratified by the occurrence of major adverse cardiovascular events (MACE) are presented in Table 2. Patients who experienced MACE had significantly higher hepatic steatosis severity, as indicated by a higher mean controlled attenuation parameter (CAP) compared with those without MACE (266.5 ± 49.3 vs. 227.9 ± 61.4 dB/m; *p* = 0.004). In parallel, the prevalence of metabolic dysfunction–associated fatty liver disease (MAFLD) was markedly higher in the MACE group than in the non-MACE group (79.2% vs. 43.8%; *p* = 0.002).

**Table 2:** Laboratory and hepatic characteristics according to the occurrence of major adverse cardiovascular events (MACE).

Characteristics	Total (*n* = 168)	MACE (*n* = 24)	Non-MACE (*n* = 144)	*p* value
Eosinophils, cells/μL	200 (100–390)	205 (65–307.5)	200 (100–397.5)	0.476^§^
Fasting plasma glucose, mmol/L	6.0 ± 1.7	5.9 ± 0.9	6.0 ± 1.8	0.832^€^
HbA1c, %	6.0 ± 0.9	6.2 ± 0.5	5.9 ± 0.9	0.307^€^
Albumin, g/L	42.0 ± 3.0	42.0 ± 2.4	42.0 ± 3.1	0.997^€^
Total protein, g/L	74.3 ± 5.1	73.2 ± 4.5	74.5 ± 5.2	0.269^€^
Total cholesterol, mmol/L	5.0 (3.8–5.9)	5.1 (3.2–6.8)	4.9 (3.9–5.8)	0.974^§^
Triglycerides, mmol/L	1.5 (1.1–2.0)	1.4 (1.1–1.8)	1.5 (1.0–2.0)	0.435^§^
HDL cholesterol, mmol/L	1.4 (1.1–1.5)	1.4 (1.2–1.6)	1.4 (1.1–1.5)	0.571^§^
LDL cholesterol, mmol/L	3.0 (2.3–3.7)	2.7 (1.9–4.3)	3.0 (2.3–3.6)	0.837^§^
CRP-hs, mg/L	3.6 (1.3–6.1)	4.2 (2.0–4.3)	3.5 (1.3–6.7)	0.164^§^
Troponin I-hs, ng/L	1.8 (0.9–3.4)	2.4 (1.2–3.8)	1.6 (0.8–3.4)	0.312^§^
Fasting insulin, μU/mL	7.3 (4.7–12.0)	8.2 (5.5–12.2)	7.3 (4.7–11.9)	0.532^§^
HOMA-IR	1.8 (1.1–3.3)	2.1 (1.3–3.5)	1.8 (1.1–3.1)	0.348^§^
CAP, dB/m	233.4 ± 61.2	266.5 ± 49.3	227.9 ± 61.4	**0.004** ^€^
Liver stiffness, kPa	4.9 (3.9–5.9)	5.1 (4.0–6.6)	4.8 (3.9–5.8)	0.387^§^
Significant fibrosis (F2–F4), *n* (%)	19 (11.4)	5 (20.8)	14 (9.7)	0.155^‡^
MAFLD, *n* (%)	82 (48.8)	19 (79.2)	63 (43.8)	**0.002** ^†^

Data are presented as mean ± SD, median (interquartile range), or *n* (%), as appropriate.

^†^*p* value calculated using the Chi-square test.

^‡^*p* value calculated using Fisher’s exact test.

^§^*p* values calculated using Mann–Whitney *U* test.

^€^*p* values calculated using Student’s *t*-test.

CAP, controlled attenuation parameter; CRP-hs, high-sensitivity C-reactive protein; HOMA-IR, homeostasis model assessment of insulin resistance; MAFLD, metabolic dysfunction–associated fatty liver disease.

Bold values indicate statistically significant *p* values (*p* < 0.05).

No significant differences were observed between groups with respect to eosinophil count, fasting plasma glucose, HbA1c, serum albumin, total protein, lipid profile (total cholesterol, triglycerides, HDL- and LDL-cholesterol), high-sensitivity C-reactive protein, high-sensitivity troponin I, fasting insulin levels, or HOMA-IR index (all *p* > 0.05). Liver stiffness measurements and the prevalence of significant liver fibrosis (F2–F4) were also comparable between patients with and without MACE.

### 3.1 Event-free survival according to MAFLD status

**Figure 2:**
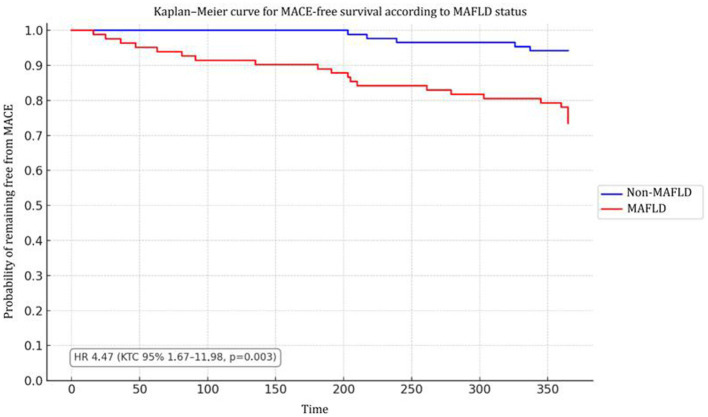
Kaplan–Meier curves showing the probability of remaining free from MACE during 12-month follow-up in patients with and without MAFLD.

During the 12-month follow-up, patients with MAFLD had significantly shorter MACE-free survival than those without MAFLD (321.9 ± 10.9 vs. 359.2 ± 2.9 days), representing an absolute between-group difference of approximately 38 days (Table 3). Kaplan–Meier analysis showed a significantly lower probability of remaining free from major adverse cardiovascular events in the MAFLD group (log-rank *p* = 0.001), with earlier decline of the survival curve over time. Consistently, in univariable Cox proportional hazards analysis, baseline MAFLD was associated with a more than fourfold higher risk of incident MACE (HR 4.47, 95% CI 1.67–11.98), further supporting its association with adverse cardiovascular outcomes in patients with stable COPD (Figure 2).

**Table 3:** Mean MACE-free survival time during 12-month follow-up according to MAFLD status.

Group	*n*	MACE-free survival time (days)		*p* value
		Mean	Standard deviation	
MAFLD	82	321.9	10.9	0.001^θ^
Non-MAFLD	86	359.2	2.9	
Total	168	340.9	5.7	

^θ^*p* value calculated using the log-rank test.

These longitudinal findings should also be interpreted in the context of our previously published baseline cross-sectional analysis of the same stable COPD population. In that baseline study, 48.8% of patients met the diagnostic criteria for MAFLD (8), and patients with MAFLD had greater dyspnoea burden, worse lung function, more frequent exacerbations, and a higher prevalence of several metabolic abnormalities compared with those without MAFLD (8). Taken together, these baseline differences support the view that MAFLD in COPD reflects a broader cardiometabolic and symptom burden that may contribute to subsequent cardiovascular vulnerability.

**Figure 3:**
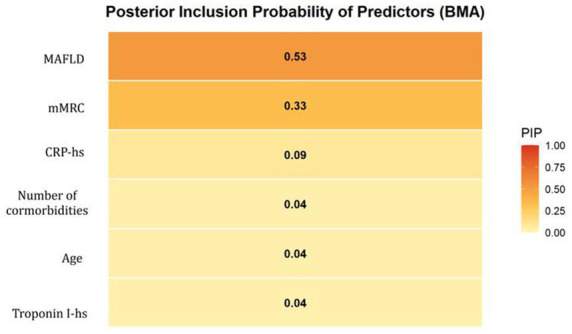
Posterior inclusion probabilities of candidate predictors derived from Bayesian Model Averaging.

To identify the most informative set of predictors for MACE and to account for model uncertainty, Bayesian Model Averaging (BMA) was applied to all prespecified candidate variables (Figure 3). Bayesian Model Averaging (BMA) demonstrated substantial heterogeneity in the relative importance of candidate predictors for major adverse cardiovascular events.

Among all variables evaluated, MAFLD showed the highest posterior inclusion probability (PIP = 0.53), indicating strong and consistent posterior support. Symptom burden assessed by the modified Medical Research Council (mMRC) scale ranked second (PIP = 0.33). In contrast, systemic inflammation (hs-CRP), myocardial injury (troponin I-hs), age, and the number of comorbidities showed low posterior inclusion probabilities (all PIP ≤ 0.1), suggesting limited and unstable contributions across the Bayesian model space.

Posterior mean odds ratios and corresponding 95% credible intervals for candidate predictors derived from Bayesian Model Averaging are summarized in Table 4, with MAFLD showing the highest posterior mean odds ratio.

**Table 4:** Posterior mean odds ratios and 95% credible intervals of candidate predictors for 12-month major adverse cardiovascular events derived from BMA.

Rank	Predictor	Posterior mean OR^a^	95% credible interval^a^
1	MAFLD	2.20	0.42–11.54
2	mMRC score	1.33	0.54–3.30
3	CRP-hs	1.00	0.97–1.04
4	Number of comorbidities	0.997	0.92–1.08
5	Age	1.00	0.99–1.01
6	Troponin I-hs	1.00	0.99–1.00

a Odds ratios represent posterior mean estimates obtained from Bayesian Model Averaging. Credible intervals reflect uncertainty across the Bayesian model space.

**Figure 4:**
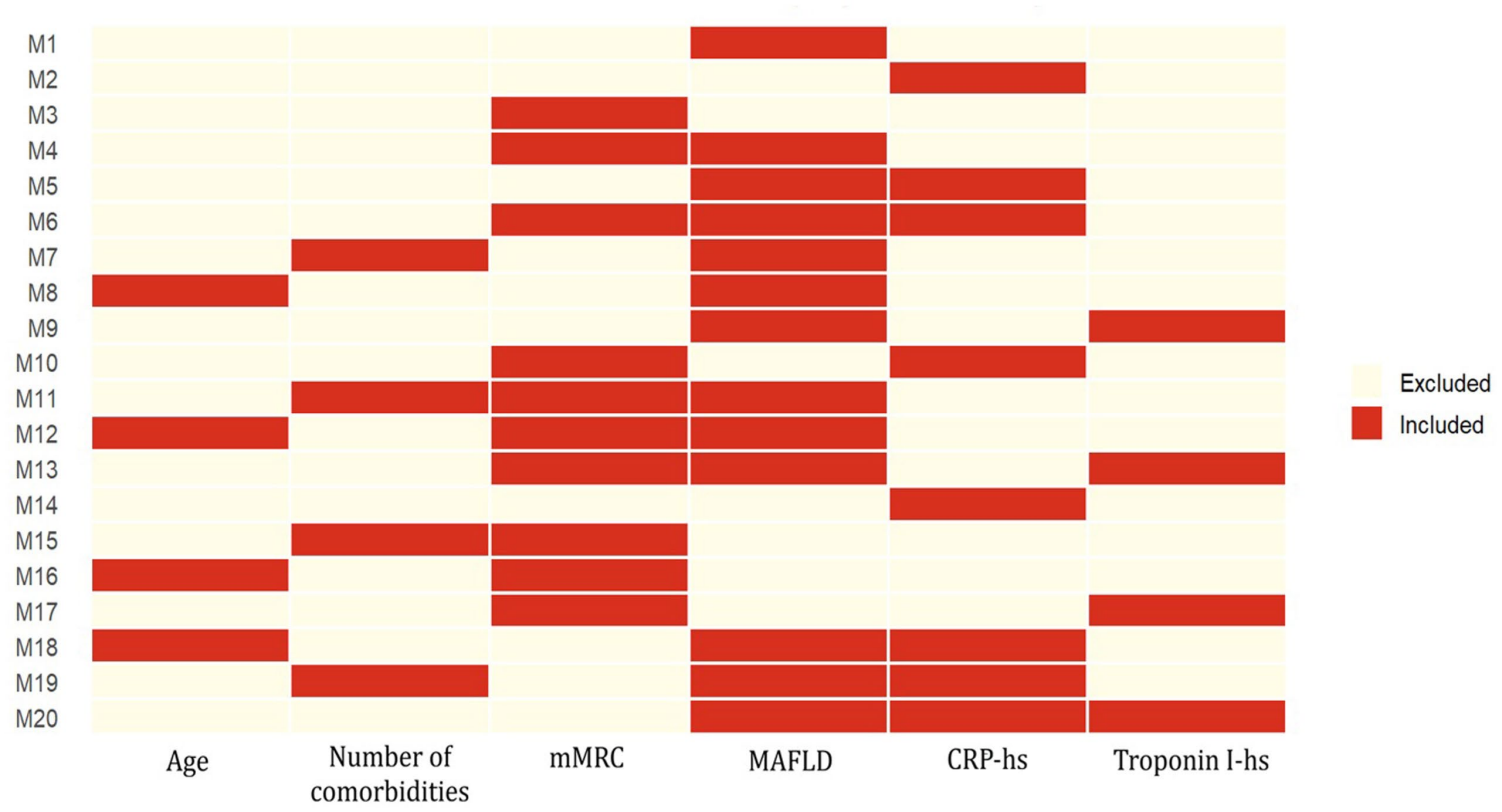
BMA model inclusion matrix for the top 20 models. Rows represent individual models ranked by posterior probability, and columns indicate candidate predictors. Red cells denote included predictors, while light cells indicate exclusion, illustrating model uncertainty and predictor stability across the model space.

The structure of the Bayesian model space and the consistency of candidate predictors across the highest-ranking models are illustrated in Figure 4.

The model inclusion matrix illustrates the composition of the top 20 Bayesian models. MAFLD and mMRC appear consistently across high-ranking models, whereas age, comorbidity count, hs-CRP, and troponin I-hs show greater variability, reflecting differential posterior support among candidate predictors.

Model selection: The highest posterior probability model (HPM) identified by Bayesian Model Averaging included MAFLD as the sole predictor. Although this model was statistically supported within the Bayesian framework, its discriminative performance and clinical informativeness were limited. Among the models with strong Bayesian support, a multivariable model incorporating MAFLD, mMRC score, and hs-CRP demonstrated superior discrimination, achieving the highest area under the receiver operating characteristic curve (AUC ≈ 0.75). Accordingly, this model was selected for subsequent nomogram development, balancing Bayesian evidence, predictive performance, and clinical interpretability.

**Figure 5:**
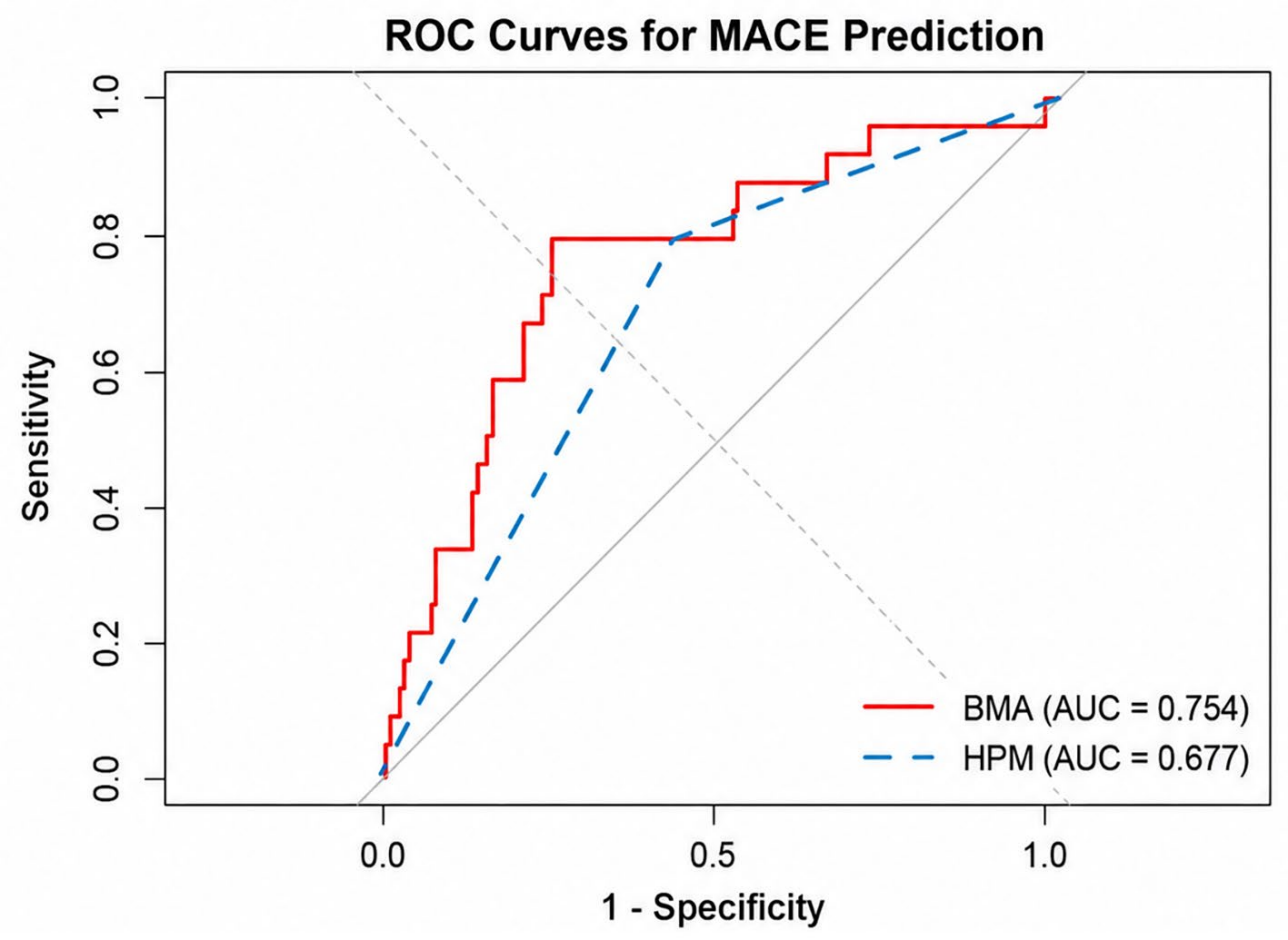
Receiver operating characteristic (ROC) curves comparing the highest posterior probability model and the selected BMA—supported model for prediction of 12-month major adverse cardiovascular events. The red solid line represents the multivariable model selected from Bayesian Model Averaging (MAFLD, mMRC score, and CRP-hs), which demonstrated superior discriminative performance (AUC = 0.754). The blue dashed line represents the highest posterior probability model (MAFLD alone), which showed lower discrimination (AUC = 0.677). The diagonal grey line indicates no-discrimination.

Discrimination: The selected BMA-supported model showed good discrimination for predicting MACE, with an AUC of approximately 0.75 on ROC analysis (Figure 5).

**Figure 6:**
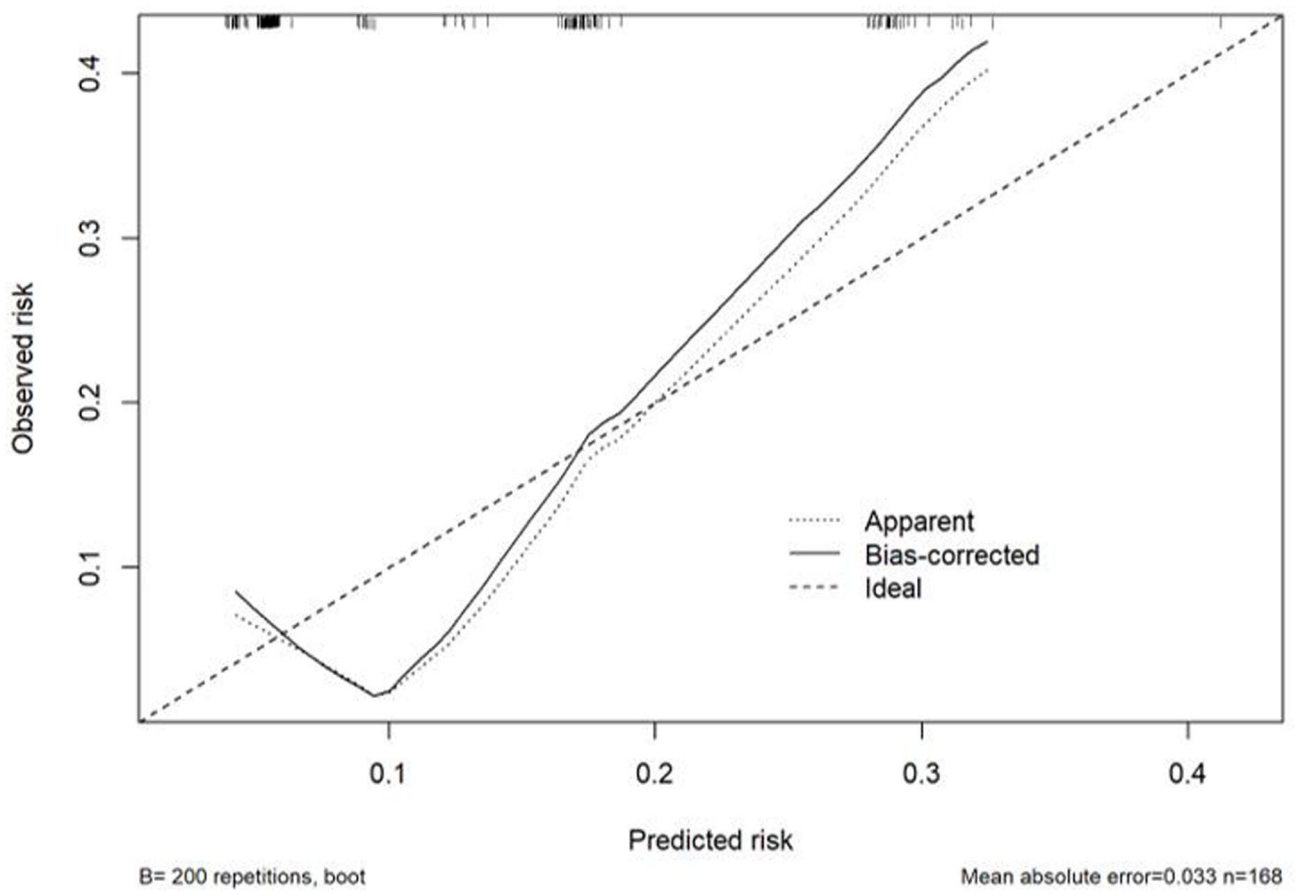
Calibration plot of the BMA-supported model for prediction of 12-month major adverse cardiovascular events (MACE). Observed risks are plotted against predicted probabilities. The dashed line represents ideal calibration, the dotted line shows apparent calibration, and the solid line indicates bootstrap bias-corrected calibration. Overall agreement between predicted and observed risks was good, with slight underestimation at higher predicted risk levels.

Calibration: Bootstrap calibration demonstrated good overall agreement between predicted and observed risks. The mean absolute error was 0.033, and the mean squared error was 0.00196. Logistic recalibration yielded a calibration intercept of 1.52 and a slope of 1.92, suggesting slight miscalibration at higher predicted risk levels, where the model tended to underestimate absolute risk, while maintaining adequate discrimination across the risk spectrum (Figure 6).

**Figure 7:**
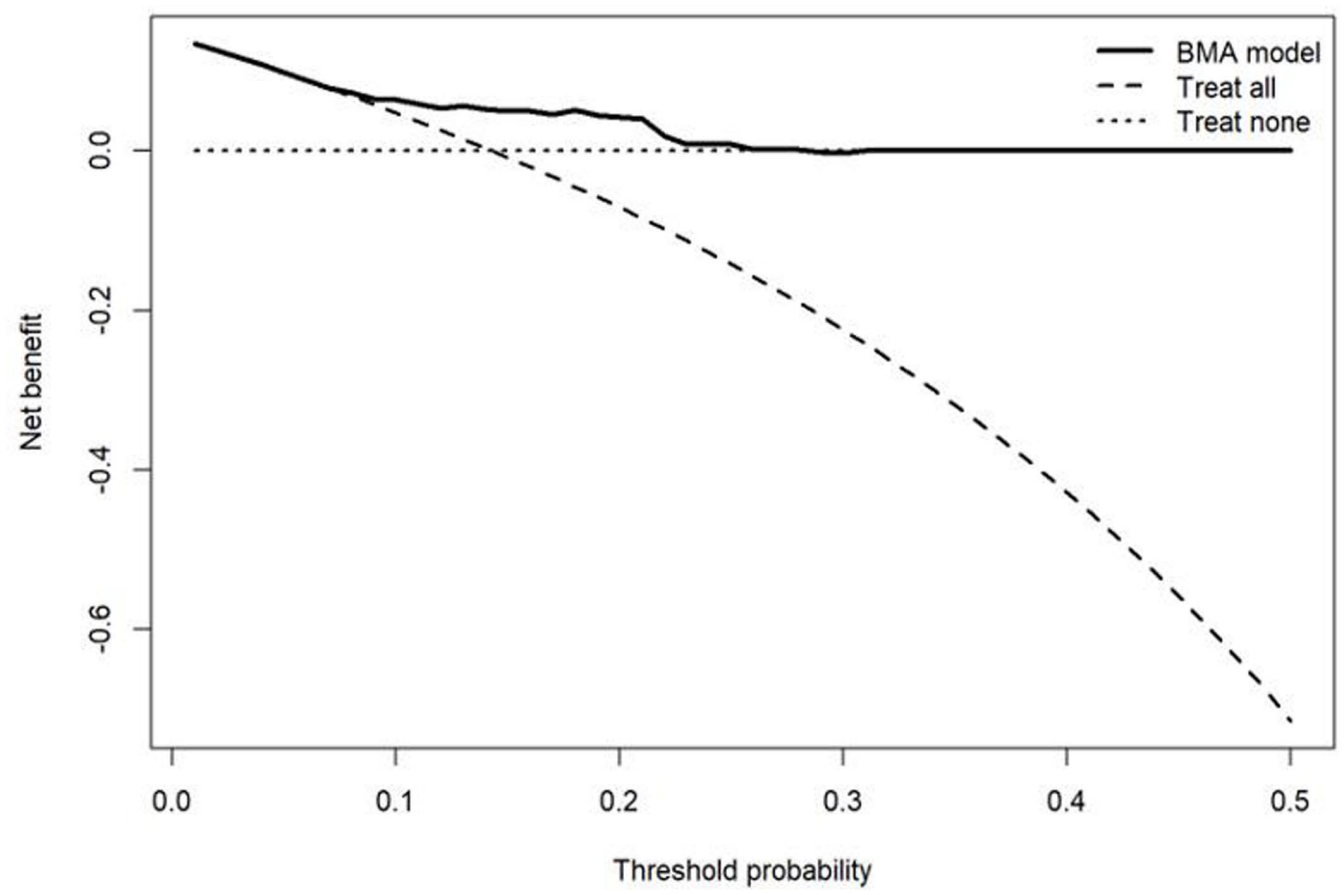
Decision curve analysis of the BMA-selected model for predicting 12-month major adverse cardiovascular events (MACE). The solid line represents the net benefit of the BMA-selected prediction model, while the dashed and dotted lines represent the treat-all and treat-none strategies, respectively. The model demonstrates superior net benefit across a wide range of threshold probabilities, indicating favorable clinical utility compared with default strategies.

Decision curve analysis: Decision curve analysis demonstrated that the BMA-selected prediction model provided a consistently higher net benefit than both the treat-all and treat-none strategies across a clinically relevant range of threshold probabilities. The net benefit of the model was particularly evident at low-to-moderate threshold probabilities (approximately 5–30%), which are commonly encountered in clinical decision-making for cardiovascular risk stratification. These findings indicate that applying the model to guide clinical decisions would result in improved identification of patients at high risk of MACE, while minimizing unnecessary interventions in low-risk individuals, thereby supporting the potential clinical utility of the proposed model (Figure 7).

### 3.2 Nomogram development and performance

**Figure 8:**
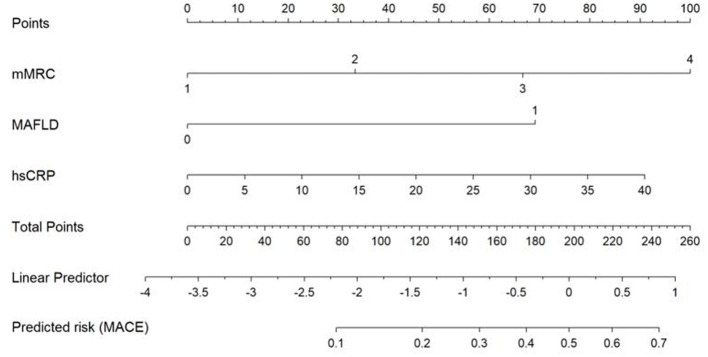
Nomogram for predicting 12-month major adverse cardiovascular events (MACE) in patients with stable COPD. The nomogram incorporates the mMRC dyspnoea score, presence of MAFLD, and CRP-hs level. For each variable, a vertical line is drawn to the “Points” axis to determine the corresponding score. The sum of points is projected onto the “Total Points” axis, which is then translated into an individualized predicted probability of MACE in 12 months.

A nomogram was constructed based on the final BMA-supported multivariable logistic regression model incorporating the modified Medical Research Council (mMRC) dyspnoea score, metabolic dysfunction–associated fatty liver disease (MAFLD), and high-sensitivity C-reactive protein (hs-CRP). Each predictor was assigned a weighted point value proportional to its regression coefficient, allowing estimation of an individual patient’s total risk score. The sum of points was then mapped to the corresponding predicted probability of 12-month major adverse cardiovascular events (MACE) (Figure 8).

## 4 Discussion

During the 12-month follow-up of patients with stable COPD derived from our previously published Vietnamese Journal of Medicine (8), we observed that 14.3% of participants experienced at least one major adverse cardiovascular event (MACE). Among the recorded cardiovascular events, acute myocardial infarction was the most frequent manifestation, accounting for 45.8% of all MACE cases. Other events, including coronary revascularization, hospitalization for heart failure, stroke, and all-cause mortality, were also observed. This finding is consistent with current evidence in the Global Initiative for Chronic Obstructive Lung Disease (GOLD) 2025 report (1), which emphasizes cardiovascular disease as one of the most prevalent and clinically relevant comorbidities in patients with COPD, substantially contributing to morbidity and mortality.

In the comparison of baseline clinical characteristics according to the occurrence of MACE, several disease-related variables differed significantly between groups. Patients who developed MACE had a significantly higher symptom burden, as reflected by higher CAT scores compared with those without MACE (19.9 ± 5.5 vs. 17.1 ± 5.4; *p* = 0.018). In addition, a history of frequent exacerbations (≥2 moderate or severe exacerbations in the preceding year) was more common in the MACE group (58.3% vs. 34.7%; *p* = 0.040), suggesting that exacerbation-prone COPD phenotypes may carry an increased cardiovascular risk. Furthermore, the distribution of GOLD symptom–risk groups differed significantly between groups (*p* = 0.044), with a markedly higher proportion of patients classified as GOLD group E among those experiencing MACE (75.0% vs. 47.2%). These findings indicate that symptom severity and exacerbation burden, rather than airflow limitation alone, are closely associated with cardiovascular outcomes in stable COPD.

Regarding laboratory and hepatic characteristics, patients who experienced MACE exhibited significantly more severe hepatic steatosis, as reflected by a higher mean controlled attenuation parameter (CAP) compared with those without MACE (266.5 ± 49.3 vs. 227.9 ± 61.4 dB/m; *p* = 0.004). In parallel, the prevalence of metabolic dysfunction–associated fatty liver disease (MAFLD) was markedly higher in the MACE group than in the non-MACE group (79.2% vs. 43.8%; *p* = 0.002). In contrast, liver stiffness measurements and the prevalence of significant liver fibrosis (F2–F4) did not differ significantly between groups, suggesting that hepatic steatosis and metabolic liver dysfunction, rather than advanced fibrosis, are more closely associated with short-term cardiovascular risk in stable COPD. Other metabolic and inflammatory biomarkers, including glucose indices, lipid profile, hs-CRP, troponin I-hs, and HOMA-IR, were comparable between groups. These findings support the concept that MAFLD and steatosis severity capture an integrated cardiometabolic risk signal.

Time-to-event analysis further reinforced these observations. Kaplan–Meier analysis demonstrated a significantly shorter mean MACE-free survival time in patients with MAFLD compared with those without MAFLD, with an absolute difference of approximately 38 days over 12 months (321.9 ± 10.9 days vs. 359.2 ± 2.9 days; *p* = 0.001). The survival curves showed an earlier and more pronounced decline in MACE-free probability in the MAFLD group throughout follow-up. Consistently, Cox proportional hazards analysis revealed that the presence of MAFLD was associated with a more than fourfold increased risk of incident MACE (Hazard ratio 4.47; 95% confidence interval 1.67–11.98; *p* = 0.003).

Our findings are concordant with emerging evidence highlighting the multisystem nature of MAFLD and its relevance to cardiovascular risk. A recent review on metabolic dysfunction–associated steatotic liver disease emphasized that MAFLD is highly prevalent among individuals with metabolic abnormalities and is closely linked to adverse cardiometabolic outcomes, including increased cardiovascular morbidity and mortality (17). Although the SAGE review does not specifically quantify cardiovascular event rates in COPD populations, it underscores shared pathogenic mechanisms such as low-grade systemic inflammation, insulin resistance, and oxidative stress that contribute to both hepatic steatosis and cardiovascular disease. These mechanisms are consistent with the biological pathways implicated in COPD, where chronic inflammation and metabolic dysregulation may interact to amplify cardiovascular risk. This recognition supports our observation that COPD patients with MAFLD experienced significantly higher incidence of major adverse cardiovascular events within 1 year of follow-up (17). Furthermore, the review call for integrated screening and risk stratification in patients with respiratory and metabolic comorbidities resonates with our clinical approach. Within the context of MAFLD, alongside traditional risk assessment tools, a more comprehensive assessment of cardiovascular issues in COPD patients can be achieved. The earlier detection and management of metabolic dysfunction and fatty liver in COPD patients could therefore offer opportunities for more holistic risk reduction strategies.

Our findings are partly concordant with those reported by Viglino et al. (18), who investigated hepatic involvement in COPD using non-invasive serum-based tools such as SteatoTest, NashTest, and FibroTest. In that study, liver fibrosis—rather than hepatic steatosis or non-alcoholic steatohepatitis—emerged as the only significant predictor of cardiovascular events and mortality, with patients exhibiting fibrosis experiencing nearly a threefold increased risk (HR 2.94; 95% CI 1.18–7.33). In contrast, steatosis alone was not associated with adverse cardiovascular outcomes. A key distinction between the two studies lies in the disease classification strategy. While Viglino et al. (18) stratified patients according to the presence of liver fibrosis, our study focused on MAFLD, a construct that encompasses hepatic steatosis in conjunction with metabolic dysfunction, including insulin resistance, dyslipidaemia, obesity, systemic inflammation, and impaired glucose metabolism. This difference suggests that the combination of fatty liver and metabolic derangements—rather than hepatic fat accumulation alone—may represent the principal mechanism driving excess cardiovascular risk in COPD.

Additionally, the research reinforces that MAFLD is a multisystem disease with significant extra-hepatic consequences (19), notably cardiovascular risk, and supports the clinical significance of incorporating MAFLD assessment into cardiovascular risk prediction models for COPD populations.

A key methodological strength of the study lies in the application of Bayesian Model Averaging (BMA) to explicitly address model uncertainty. Unlike conventional stepwise selection approaches that rely on a single “best” multivariable model, BMA integrates evidence across the entire space of plausible models and quantifies the relative importance of candidate predictors through posterior inclusion probabilities (PIP), thereby providing a more robust and transparent framework for variable selection.

Within the Bayesian model space, MAFLD demonstrated the highest posterior inclusion probability (PIP = 0.53), indicating the most consistent and stable posterior support as a predictor of 12-month major adverse cardiovascular events (MACE). Symptom burden, as assessed by the modified Medical Research Council (mMRC) dyspnoea scale, ranked second (PIP = 0.33), whereas systemic inflammation (hs-CRP), age, number of comorbidities, and myocardial injury indexed by high-sensitivity troponin I all showed low posterior inclusion probabilities (PIP < 0.10), suggesting limited and unstable contributions across competing models. These findings suggest that MAFLD was the most consistently supported predictor within the Bayesian model space and, to a lesser extent, dyspnoea severity in cardiovascular risk stratification among patients with stable COPD.

The highest posterior probability model (HPM) identified by BMA included MAFLD as the sole predictor. Although this model was statistically supported within the Bayesian framework, its discriminative performance was modest (AUC ≈ 0.68), underscoring the limitations of relying exclusively on posterior probability when selecting clinically useful prediction models. This observation illustrates an important practical consideration in Bayesian prediction research: models with strong posterior support may still be overly parsimonious and insufficient for accurate individual risk discrimination.

To balance Bayesian evidence with predictive performance and clinical interpretability, an additional model selection step was therefore undertaken. Among models with substantial posterior support, a multivariable model incorporating MAFLD, mMRC score, and hs-CRP showed good discrimination among Bayesian-supported models (AUC ≈ 0.75) and was selected for nomogram development. This approach allowed retention of the strongest Bayesian signal (MAFLD) while incorporating clinically meaningful dimensions of symptom burden and systemic inflammation, thereby enhancing model performance without excessive complexity.

Beyond discrimination, the clinical value of a prediction model critically depends on calibration and decision-analytic performance. In this study, bootstrap calibration demonstrated good overall agreement between predicted and observed risks, with low mean absolute and squared errors, supporting the internal validity of the BMA-selected model. Although recalibration indicated a slope greater than unity and an intercept shift—suggesting mild underestimation of absolute risk at higher predicted probabilities—this pattern is commonly observed in moderate-sized cohorts and does not negate the model’s overall reliability across the clinically relevant risk range. Importantly, the preservation of adequate discrimination despite minor miscalibration suggests that the model remains informative for risk stratification rather than precise absolute risk estimation, which can be further refined through external validation.

Decision curve analysis provides complementary insight into the potential clinical utility of the model. Across a broad range of threshold probabilities, particularly within the low-to-moderate range (approximately 5–30%), the BMA-selected model consistently achieved a higher net benefit than both treat-all and treat-none strategies. This finding is clinically meaningful, as cardiovascular preventive decisions in patients with stable COPD are often made within this probability spectrum, where overtreatment and undertreatment represent competing concerns. The favourable decision curves indicate that incorporating the model into clinical decision-making could improve identification of patients at higher cardiovascular risk while minimizing unnecessary interventions in lower-risk individuals. This finding should be interpreted cautiously given the exploratory nature of the model and the absence of external validation.

The nomogram derived from the final BMA-supported multivariable model translates these findings into an exploratory risk estimation framework. By integrating MAFLD status, symptom burden quantified by the mMRC dyspnoea scale, and systemic inflammation reflected by hs-CRP, the nomogram enables individualized estimation of 12-month MACE risk using readily available clinical and laboratory parameters. The relative weighting of predictors in the nomogram mirrors their posterior importance within the Bayesian framework, reinforcing the central role of metabolic–hepatic dysfunction alongside respiratory symptom severity and inflammation in shaping cardiovascular risk among patients with stable COPD.

Overall, the calibration, decision curve, and nomogram analyses indicate that the proposed model provides additional information that may be useful in clinical practice. Whereas cardiovascular risk assessment in patients with stable COPD has traditionally relied largely on clinical judgement, the proposed nomogram offers an additional, structured tool to support risk stratification and risk estimation, particularly in patients with concomitant MAFLD.

From a conceptual perspective, our findings suggest that cardiovascular risk in this cohort is more strongly driven by cardiometabolic dysfunction than by traditional measures of COPD severity. In particular, MAFLD consistently emerged as the most robust and stable predictor across multiple analytical approaches. This interpretation aligns with current understanding that outcomes in COPD are substantially influenced by comorbid conditions, particularly metabolic and inflammatory disorders.

### 4.1 Clinical implications

The proposed BMA-supported model and nomogram may provide a preliminary framework for cardiovascular risk stratification in patients with stable COPD. By integrating MAFLD, mMRC score, and hs-CRP, the model highlights the potential contribution of metabolic and symptom-related factors beyond conventional risk assessment and may help increase clinical awareness of MAFLD in this population. Nevertheless, in view of the limited number of events and the absence of external validation, the model should be considered exploratory and requires further validation before any clinical application.

### 4.2 Strengths and limitations

This study has several strengths. First, the prospective 12-month follow-up allowed systematic ascertainment of major adverse cardiovascular events in patients with stable COPD. Second, MAFLD was diagnosed using transient elastography (FibroScan) in combination with metabolic biomarkers, providing an objective and comprehensive assessment of metabolic liver disease beyond conventional clinical definitions. Third, the application of Bayesian Model Averaging enabled explicit handling of model uncertainty and supported robust predictor selection, complemented by calibration analysis and decision curve analysis to evaluate model performance and potential clinical utility.

Several limitations should be acknowledged. The mMRC scale reflects overall dyspnoea burden but does not differentiate pulmonary from cardiovascular contributions to breathlessness. In addition, baseline coronary symptoms and subclinical coronary artery disease were not systematically characterized, which may have introduced some residual confounding related to underlying cardiovascular status. The relatively small number of outcome events and the single-center design may also have influenced the stability and generalizability of the model. Nevertheless, internal bootstrap validation showed acceptable performance within the present cohort. Further evaluation in independent COPD populations will be important to confirm the reproducibility and broader applicability of the model.

## 5 Conclusion

In conclusion, baseline MAFLD was associated with a higher risk of 12-month major adverse cardiovascular events in patients with stable COPD, underscoring the relevance of cardiometabolic comorbidity in risk stratification. In an exploratory analysis, a Bayesian Model Averaging–based model incorporating MAFLD, mMRC, and hs-CRP demonstrated acceptable internal performance for short-term risk prediction. These findings support greater clinical attention to MAFLD in COPD and warrant further investigation, with external validation required before individualized risk prediction can be applied.

## Data Availability

The datasets presented in this article are not readily available because the datasets generated and/or analyzed during the current study are not publicly available due to institutional and ethical restrictions but are available from the corresponding author upon reasonable request, subject to approval by the relevant ethics committees. Requests to access the datasets should be directed to HanhDoan, minhhanh2324@gmail.com.
